# The role of prostate-specific antigen in the osteoblastic bone metastasis of prostate cancer: a literature review

**DOI:** 10.3389/fonc.2023.1127637

**Published:** 2023-09-07

**Authors:** Xu Zhang, Peng Jiang, Chaojun Wang

**Affiliations:** Department of Urology, the First Affiliated Hospital, Zhejiang University School of Medicine, Hangzhou, Zhejiang, China

**Keywords:** prostate-specific antigen, osteoblastic bone metastasis, osteoclast, osteoblast, insulin-like growth factor, transforming growth factor β2, parathyroid hormone-related protein

## Abstract

Prostate cancer is the only human malignancy that generates predominantly osteoblastic bone metastases, and osteoblastic bone metastases account for more than 90% of osseous metastases of prostate cancer. Prostate-specific antigen (PSA) plays an important role in the osteoblastic bone metastasis of prostate cancer, which can promote osteomimicry of prostate cancer cells, suppress osteoclast differentiation, and facilitate osteoblast proliferation and activation at metastatic sites. In the meantime, it can activate osteogenic factors, including insulin-like growth factor, transforming growth factor β2 and urokinase-type plasminogen activator, and meanwhile suppress osteolytic factors such as parathyroid hormone-related protein. To recapitulate, PSA plays a significant role in the osteoblastic predominance of prostate cancer bone metastasis and bone remodeling by regulating multiple cells and factors involved in osseous metastasis.

## 1 Introduction

Prostate cancer (PCa) is the only human malignancy that generates predominantly osteoblastic bone metastases. Bone metastasis is one of the hallmarks of PCa progression, mainly presenting as osteoblastic (>90%), followed by mixed (namely osteoblastic plus osteoclastic, <10%) and rarer osteoclastic metastatic lesions (1, 2). Current evidences suggest that malignant tumor cells accelerate bone remodeling at metastatic sites via secreting multiple osteolytic factors, such as parathyroid hormone-related protein (PTHrP), interleukin-6 (IL-6) and tumor necrosis factor (TNF), thereby stimulating the proliferation and differentiation of osteoclasts (OC) and potentiating osteolytic processes (3, 4). This on one hand provides space for metastatic cancer cell colonization and secondary nascent bone formation (5, 6). On the other hand, it releases the intrinsic growth/osteogenic factors in bone matrix, including abundant quantities of insulin-like growth factor (IGF) and transforming growth factor β2 (TGFβ2), and relatively lower levels of bone morphogenetic protein (BMP), platelet-derived growth factor (PDGF) and fibroblast growth factor (FGF), to stimulate the proliferation of metastatic cancer cells and activate osteoblasts (OB), hence accelerating osseous metastasis and inducing pathological nascent bone formation (3, 7).

During the bone remodeling induced by other malignant tumors except PCa, the osteolytic effects of OCs usually outperform the osteogenic effects of secondarily activated OBs; therefore, most malignancies except for PCa present osteoclastic bone metastases (8, 9). Nevertheless, as for PCa, the existence of a prostate-specific factor, namely prostate-specific antigen (PSA), contributes greatly to the osteoblastic predominance of PCa bone metastasis. PSA produced by tumor cells can promote the phenotypic switch of PCa cells towards OB phenotype (10, 11). Meanwhile, PSA can inhibit OC differentiation and facilitate OB proliferation and activation at metastatic sites. Additionally, PSA may also advance osteogenic responses by activating IGF and TGFβ2 at metastatic sites. These functions of PSA lead to the proliferation and activation of OBs, making their secondary osteogenic effects outweigh the osteolytic effects of OCs during PCa bone remodeling, and thus render PCa bone metastases predominantly osteoblastic. To our knowledge, no literature has hitherto systemically summarized how PSA exerts its influences in the osteoblastic bone metastasis of PCa, and we herein present a review on this issue.

## 2 Structure and activation of PSA

PSA belongs to the human kallikrein family of serine proteases, categorized as plasma kallikrein (gene located at human chromosome 4q35) and tissue kallikrein (gene located at chromosome 19q13-14). The tissue kallikrein subfamily genes contain 15 members named KLK1-KLK15, and the corresponding proteins are hK1-hK15. KLK1/2/3 encode pancreatic/renal kallikrein (hK1), human glandular kallikrein (hK2) and prostate-specific antigen (hK3, namely PSA), respectively. These three proteins share approximately 80% structural homology with a kallikrein ring formed by five disulfide bonds and are known as the typical kallikrein proteins. hK2 and hK3 (PSA) are only expressed by prostate cells, and PSA can be activated by hK2, hK4 and hK15 (12).

**Figure 1:**
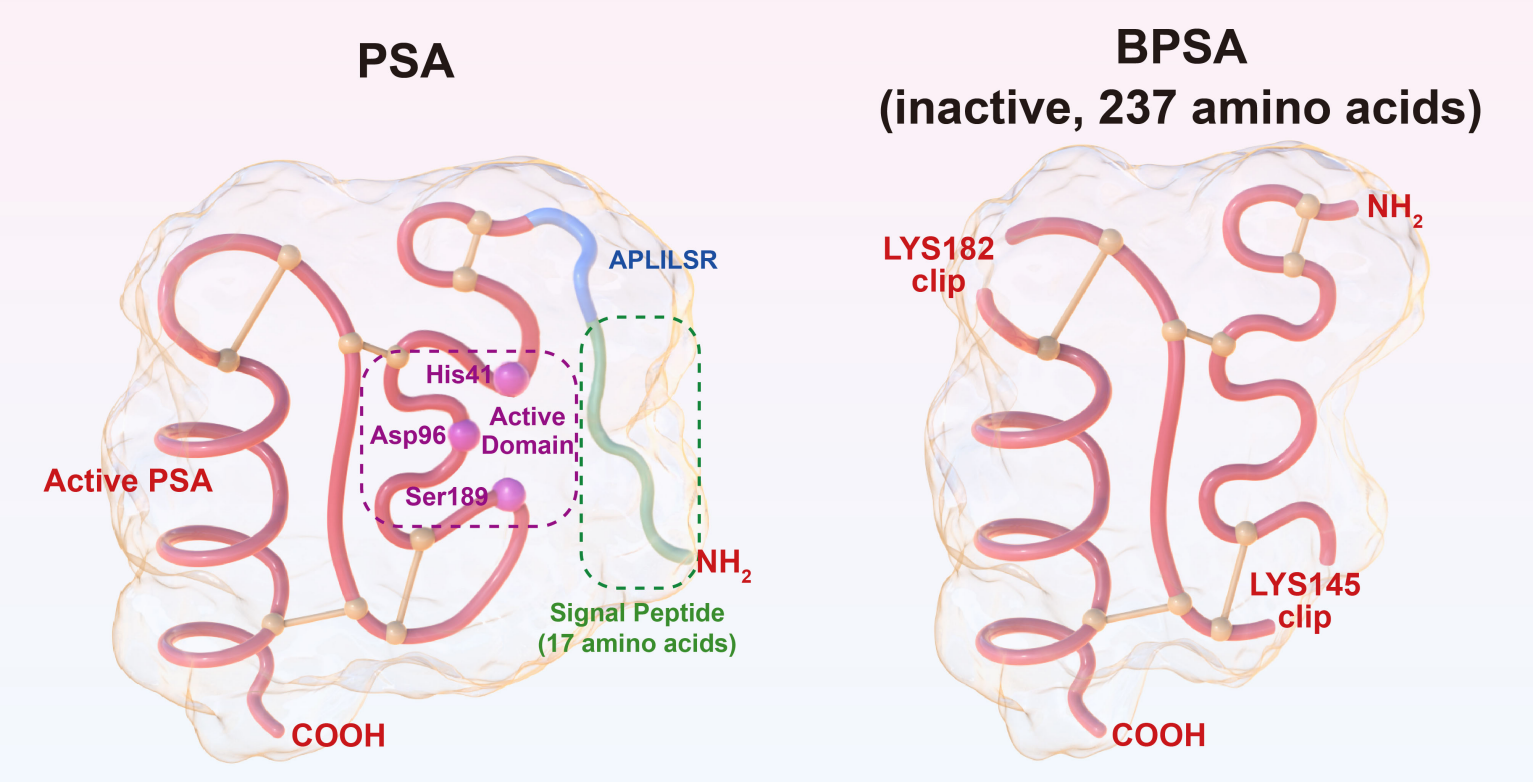
Illustration of the structures of PSA and BPSA.

PSA is a single-chain polypeptide with a molecular weight of 33-34 kD containing 237 amino acids. It is obtained by hydrolyzing the N-terminal, 17-amino acid signal peptide and the 7-amino acid “APLILSR” segment off the 261-amino acid PSA precursor. Notably, only 237 amino acids confer activity on PSA, and PSA products with more or less than 237 amino acids are all inactive (13). Besides, there exists benign PSA (BPSA) that also contains 237 amino acids but remains inactive due to the cleavage of peptide bonds at lysine 145 and lysine 182 (14). PSA is essentially a serine protease, and the triplex catalytic domain of active PSA composed of histidine 41, aspartate 96 and serine 189 can specifically hydrolyze “HSSKLQ” peptide (15). The structures of PSA and BPSA are illustrated in **Figure 1**. PSA antagonists have been discovered in human blood, including α-anti-chymotrypsin (ACT), α2-macroglobulin (A2M), etc. It has been shown that PSA-ACT covalent binding results in complete loss of enzymatic activity of PSA; however, PSA-A2M compound retains partial enzymatic activity of PSA and is still capable of hydrolyzing “HSSKLQ” peptide, although PSA and A2M have much stronger binding, and PSA-A2M compound cannot be detected via ELISA (13). Overall, PSA precursor, BPSA and PSA-ACT complex detected in human blood are all inactive. In nonmalignant prostate tissue, owing to its serine protease activity, the main physiological function of PSA is to hydrolyze seminogelin and fibronectin present in high concentrations in seminal plasma, thus liquefying seminal clot after ejaculation, which is essential for post-ejaculatory sperm motility (16).

## 3 Elevated seral/cellular PSA levels significantly correlate with osteoblastic bone metastasis of PCa

PCa osteoblastic bone metastasis is characterized by immature nascent osteoid at metastatic sites, accompanied by disorganized collagen fibers with impaired strength and large numbers of metastatic cancer cells (17). Patients with metastases are predisposed to skeletal-related events, such as pathological fracture, pain or spinal cord compression (18). Yonou et al. reported that osteoblastic bone metastases accounted for more than 90% of osseous metastases of PCa, and PCa patients with bone metastases usually had significantly increased blood PSA levels (≥10 ng/ml in approximately 99% of patients) (2). Correspondingly, a survey by Doherty et al. of 27 PCa patients with osseous metastases demonstrated that low levels of blood PSA were significantly correlated with osteoclastic bone metastases (19). In addition, Roudier et al. conducted autopsy for 14 patients who died of PCa, and found that 12 of 14 patients presented diffuse osteoblastic metastases; further immunostaining of the osseous metastases revealed that averagely 75% of tumor cells at metastatic sites expressed immunoreactive PSA, and over 40% of the patients had over 90% of tumor cells at metastatic sites expressing immunoreactive PSA (20).

In another research, Yonou et al. grafted human adult bones into NOD/SCID mice which were injected with LNCaP and PC-3 cells through tail veins, and finally established PCa bone metastasis models. Results showed that osteoblastic or mixed metastatic tumors were formed from PSA-secreting LNCaP cells in the bone grafts, with significant formation of pathological nascent bones, large amounts of OBs and hardly spotted OCs. In the meantime, osteoclastic metastatic tumors were formed from non-PSA-secreting PC-3 cells in the bone grafts, with significantly more mature OCs on the surfaces of pathological nascent bones (21). The above studies corroborate the positive correlation between seral/cellular PSA levels and PCa osteoblastic bone metastasis.

## 4 Effects of PSA on cells involved in PCa osteoblastic bone metastasis

### 4.1 PSA can promote osteomimicry of PCa cells

Metastatic PCa cells can undergo a phenotypic switch towards an OB-like phenotype by synthesizing and secreting osteocalcin, osteopontin (22), osteoprotegerin (OPG), etc. OPG is an anti-osteolytic agent that can antagonize receptor activators of nuclear factor κB ligand (RANKL, central to OC differentiation) (23). Primary PCa tissues can also express OPG, and it has been observed that their OPG expression increases with elevated PCa grade (24). PCa cells with OB phenotype can facilitate osteogenic responses at metastatic sites, known as “osteomimicry” (10, 11). Previous studies suggested that PSA could potentiate osteomimicry of PCa cells. Stable expression of endogenous active PSA in non-PSA-secreting DU-145 cells could induce a phenotypic switch towards OB phenotype (25). Also, Chiao et al. found that PSA could induce up to a 50% increase in endothelin-1 secretion by DU-145, PC-3 and JCA-1 cells in a dose-dependent manner, and ET-1 is recognized as a strong osteogenic factor (1). Another study showed that co-culture of androgen-dependent LNCaP cells and bone stromal cells could induce the formation of androgen-independent C4→C4-2→C4-2B cell sublines step by step — with their gradually increased PSA secretion (26), their expression of osteocalcin and osteopontin was simultaneously elevated (27), and their capabilities to induce osteoblastic bone metastases in immunodeficient mice were also gradually augmented (27, 28). These results substantiate that PSA can promote the phenotypic switch of PCa cells towards OB phenotype. However, the mechanisms whereby PSA induces osteomimetic properties in PCa cells remain elusive.

### 4.2 PSA can inhibit differentiation and promote apoptosis of OCs, and suppress osteolytic responses at metastatic sites

Overwhelming evidences substantiate that most osteolytic factors, such as 1, 25-dihydroxy vitamin D3, parathyroid hormone (PTH), IL-6, etc., cannot act directly on OCs due to the absence of corresponding receptors in OC precursors, immature and mature OCs (29–31). Instead, these factors mainly act on OBs and bone stromal cells to produce RANKL and macrophage colony-stimulating factor (M-CSF). Subsequently, RANKL and M-CSF act on their receptors expressed on surfaces of OC precursors, thereby inducing OC proliferation and differentiation (6). In other words, OC differentiation, maturation and secondary osteolysis are activated by OBs, and the biological effects of most osteolytic factors are mediated by OBs (29–31). If RANKL produced by OBs is deactivated by its antibodies, the activating effects of osteolytic factors on OCs are abolished (32).

In a study by Goya et al. where differentiation of OC precursor RAW264.7 was induced with RANKL, supplementation of RANKL alone in RAW264.7 culture media led to mature OCs on day 7. However, concurrent addition of PSA with RANKL in RAW264.7 culture media resulted in few mature OCs. Meanwhile, if PSA was added in culture media two days after supplementation of RANKL, only 60-70% of RAW264.7 cells became immature OCs on day 7; but if PSA was added five days after supplementation of RANKL, the maturation process of RAW264.7 cells towards mature OCs was unaffected. This phenomenon indicated that PSA could suppress differentiation of OC precursors, but yielded no inhibitory effects on mature OCs. The study also demonstrated that PSA could induce apoptosis of OC precursor RAW264.7 in a dose-dependent manner, and the apoptosis rate could be up to 43%. Moreover, PSA-induced apoptosis of RAW264.7 cells could be significantly reduced when PSA activity was antagonized by ACT, suggesting that the enzymatic activity of PSA promoted apoptosis of OC precursors (33). In summary, PSA can inhibit differentiation and induces apoptosis of OC precursors, therefore suppressing the osteolytic effects of OCs and enhancing PCa osteoblastic bone metastasis. However, currently little is known about the molecular or biochemical mechanisms underlying how PSA acts on OCs, and further studies are warranted.

### 4.3 PSA can potentiate OB proliferation and activation and facilitate PCa osteoblastic bone metastasis

PSA can potentiate OB proliferation. Yonou et al. reported a greater than 10-fold increase of TGFβ2 expression of human osteosarcoma SaOS-2 cells (originated from osteoblasts) after the addition of exogenous active PSA in culture media, and that SaOS-2 cell proliferation increased in a dose-dependent manner (up to 124-162%); on the contrary, deactivation of PSA using antagonist ACT or anti-PSA antibodies led to 65-75% decline of SaOS-2 cell proliferation. Additionally, they revealed that direct injection of active PSA in human bone grafts of NOD/SCID mice significantly increased the number of OBs and the volume of nascent osteoid in the bone grafts, which could also be antagonized by ACT (21). These results indicate that PSA-induced OB proliferation depends on the enzymatic activity of PSA.

PSA can activate OBs and enhance their osteogenic functions. Nadiminty et al. found that after the expression of endogenous active PSA in SaOS-2 cells, levels of multiple osteogenic factors were upregulated (34). For example, crucial osteogenesis-related transcription factor RUNX2 exhibited a 25-fold increase in expression, promoting the transcription of various downstream osteogenic factors (35). The expression of other osteogenic factors, such as osteocalcin, osteopontin, TGFβ2, BMP4 and BMP8, was also upregulated. However, interestingly, central OC differentiation factor RANKL was upregulated concomitantly, whereas OPG, the antagonist of RANKL with anti-osteolytic effects, showed a 95-fold decrease in expression. This finding suggests that after the expression of PSA in SaOS-2 cells, SaOS-2 cells demonstrate not only enhanced osteogenic functions, but also strengthened capabilities to activate OCs and secondary osteolysis — this might have a subtle connection with the phenomenon that during early PCa bone metastasis, osteogenic responses are preceded by osteolytic processes which are produced by OB-activated OCs (5, 36).

In another study, Yonou et al. stimulated MG-63 and SaOS-2 cells with exogenous active PSA, which led to a greater than 3-fold increase of OPG expression in both cell lines, while RANKL expression was downregulated. The results imply that exogenous active PSA enhances the osteogenic functions of OBs but yields no effects on their capabilities to activate OCs and secondary osteolysis (2). This is exactly the opposite of the results obtained by Nadiminty et al. as described above (34). To explain this difference, Yonou et al. proposed that exogenous active PSA stimulation was more consistent with the actual conditions of PCa metastatic sites where PSA stimulates OBs, compared to intracellular expression of endogenous PSA. Besides, a recent study found that BMP4 could induce transition of endothelial cells to OBs at PCa metastatic sites, and thus another possible mechanism whereby PSA promotes PCa osteoblastic bone metastasis could be inferred: PSA upregulates BMP4 expression and secretion of OBs, thereby facilitating endothelial-OB transition at PCa metastatic sites (37). In summary, PSA may promote PCa osteoblastic bone metastasis by potentiating OB proliferation and activation and enhancing their osteogenic functions. However, the molecular or biochemical mechanisms underlying the effects of PSA on OBs require to be clarified.

## 5 Effects of PSA on factors involved in PCa osteoblastic bone metastasis

### 5.1 PSA may potentiate OB proliferation and activation through releasing active IGF (insulin-like growth factor)

IGF-I/II are the most abundant osteogenic factors in bone matrix (38) and are components of IGF system together with 2 IGF receptors and 6 IGF-binding proteins (IGFBP) distributed in tissues and blood (39). IGFs can be synthesized by PCa cells and OBs and then secreted to bone matrix. They can augment osteogenic responses by stimulating the proliferation and activation of metastatic cancer cells and OBs (40, 41). IGFBP3 is the main binding protein of IGF-I, which can function as the transport carrier of IGF-I (42). On the other hand, IGFBP3 can also block the biological effects of IGF-I via blocking its binding with IGF receptors (43). PSA can degrade IGFBP3 and then decrease its binding with IGF-I to release more active IGF-I, thus potentiating IGF-I-induced OB proliferation and activation and PCa osteoblastic bone metastasis (44). Smith et al. analyzed IGFBP3 concentration at metastatic sites and serum PSA levels of six PCa patients, and found that IGFBP3 concentration negatively correlated with PSA level (45). In addition, Miyata et al. discovered that serum IGFBP3 level and IGFBP3/PSA ratio were significantly reduced in patients with progressive PCa (46). These studies substantiate the degradative effect of PSA on IGFBP3.

### 5.2 PSA may potentiate OB proliferation and activation through activating TGFβ2 (transforming growth factor β2)

TGFβ2, synthesized by PCa cells and OBs and secreted to bone matrix, can promote proliferation and inhibit apoptosis of OBs, thereby enhancing osteogenic responses (41, 47). After being synthesized in cells, TGFβ first forms inactive “small latent” TGFβ via covalently binding with “latency-associated peptide”; then “small latent” TGFβ binds with latent TGFβ binding protein 1 (LTBP1) by a disulfide bond to form inactive “large latent” TGFβ, which is finally secreted to extracellular bone matrix and restored. To sum up, TGFβ synthesized and secreted by cells is inactive, and its activation requires the removal of LTBP1 and latency-associated peptide (48). Researchers noted that PSA could degrade the latency-associated peptide binding with TGFβ2 to accelerate the activation of TGFβ2 (49). Besides, PSA can stimulate OBs to express and secrete TGFβ2 (21, 34). Accordingly, PSA produced by metastatic PCa cells may potentiate OB proliferation and activation by facilitating the expression, secretion and activation of TGFβ2, which can further enhance PCa osteoblastic bone metastasis.

### 5.3 PSA may augment osteogenic responses at PCa metastatic sites through degrading parathyroid hormone-related protein (PTHrP) and suppressing its osteolytic effects

PTHrP is an important osteolytic factor secreted by cancer cells (41). Like PTH, it can act on cell membrane PTH/PTHrP receptors, activate adenylate cyclase and then induce downstream signal transduction (50). Importantly, PTHrP can act on OBs to increase their synthesis of RANKL and decrease their expression of OPG, thus promoting OC proliferation and differentiation (51). Moreover, PTHrP can bind with the receptors on OC surfaces and directly stimulate OC differentiation (52). During this process, the osteolytic effects of OCs usually outperform the osteogenic effects of secondarily activated OBs, and hence PTHrP secreted by cancer cells generally induces osteolytic bone metastases (8, 9). This can strongly explain the predominance of osteolytic bone metastases in breast cancer given that breast cancer cells secrete large amounts of PTHrP (53, 54). However, osteoblastic bone metastases are still predominantly observed in PCa, although metastatic PCa cells can also secrete fairly much PTHrP (55). We hypothesize that this discrepancy is attributed to PSA, which can cleave PTHrP at the position of its 23rd amino acid; the degraded and deactivated PTHrP fails to bind with PTH/PTHrP receptors on OB/OC surfaces, resulting in decreased osteolytic effects of PTHrP and relatively enhanced osteogenic processes at PCa metastatic sites (50, 56). Furthermore, Schluter et al. found that the N-terminal peptide of PTHrP cleaved by PSA could bind with and activate endothelin receptor, generating osteogenic effects similar to that of endothelin-1 (57). Therefore, PSA may augment osteogenic responses at PCa metastatic sites by degrading and deactivating PTHrP and suppressing its osteolytic effects.

### 5.4 PSA may advance PCa osteoblastic bone metastasis through activating uPA (urokinase-type plasminogen activator)

uPA, uPA receptors and 2 uPA inhibitors constitute uPA system. uPA initially synthesized in PCa cells is an inactive single-chain proenzyme, known as a single-chain urokinase-type plasminogen activator (scuPA), which can be cleaved by proteases such as plasmin to form active uPA consisting of a light chain and a heavy chain. uPA can facilitate OB proliferation and activation by binding with uPA receptors on OB surfaces (41, 58). Besides, uPA is a serine protease that can degrade extracellular matrix and promote cancer cell spreading and metastasis (59). Intriguingly, uPA produced by metastatic PCa cells can lyse bone matrix and then accelerate PCa bone metastasis and bone remodeling (60). Moreover, uPA can activate TGFβ2 precursor to further augment osteogenic responses at PCa metastatic sites (61). Taken together, these findings reveal that uPA derived from PCa cells can potently induce PCa osteoblastic bone metastasis. Yoshida et al. found that PSA could cleave scuPA at lysine 158 to generate active uPA (62). Therefore, PSA may activate uPA to advance PCa osteoblastic bone metastasis.

Interactions prevail among these PSA-influenced factors involved in PCa osteoblastic bone metastasis. For instance, IGFBP3 is also a type V TGFβ receptor ligand that can compete with TGFβ2 to bind with its receptors. Hence when PSA secreted by metastatic PCa cells degrades IGFBP3 and decreases its concentration at metastatic sites, it can be extrapolated that the osteogenic effects of TGFβ2 would be in turn enhanced (63). Furthermore, IGFBP3 can be hydrolyzed by uPA derived from metastatic PCa cells, resulting in the release of active IGF-II and enhanced osteogenic responses (64). In addition, the ability of uPA receptors to bind with IGF-II receptors further facilitates cancer cell-OB and OB-OB interactions, which subsequently promotes the proliferation of both cancer cells and OBs (65). Such interactions may further intensify the possible effects of PSA in inducing PCa osteoblastic bone metastasis.

## 6 PSA can induce the “vicious cycle” of PCa bone remodeling to be predominantly osteoblastic

Bone remodeling of malignant tumors has been acknowledged as a “vicious cycle” (3, 66). Metastatic cancer cells can secrete many osteolytic factors, such as PTHrP, IL-6, TNF (67), etc., which stimulate OC proliferation and differentiation and produce osteolytic effects. Furthermore, during the osteolytic processes, the growth/osteogenic factors stored in bone matrix, such as IGF, TGFβ2 and BMP, are released, which act back on cancer cells and OBs. On the one hand, these factors promote the proliferation of metastatic cancer cells and their synthesis and secretion of PTHrP; on the other hand, they activate OBs and induce pathological nascent bone formation. This PTHrP-IGF/TGFβ2/BMP-PTHrP positive feedback loop represents the canonical process of “vicious cycle”, which tremendously potentiates metastatic cancer cell proliferation and expedites bone remodeling at the metastatic sites.

It has been established that PSA can prevent excessive proliferation and activation of OCs by degrading and deactivating PTHrP (56, 68). Besides, PSA can activate TGFβ2 released by bone matrix and thus enable its osteogenic effects (49). Therefore, PSA can affect the “vicious cycle” of PCa bone remodeling via deactivating PTHrP and activating TGFβ2. During this process, PSA can suppress osteolysis, enhance osteogenesis, and induce predominantly osteoblastic PCa bone metastases. This can partially explain why breast cancer cells produce much PTHrP, yet most breast cancer cases present osteoclastic bone metastases (53, 54), which may be attributed to the absence of PSA in breast cancer cells. Another evidence is that in clinical practice, there exist PCa cases with low serum PSA levels who still present PTHrP-mediated osteoclastic bone metastases (50).

## 7 Discussion

**Figure 2:**
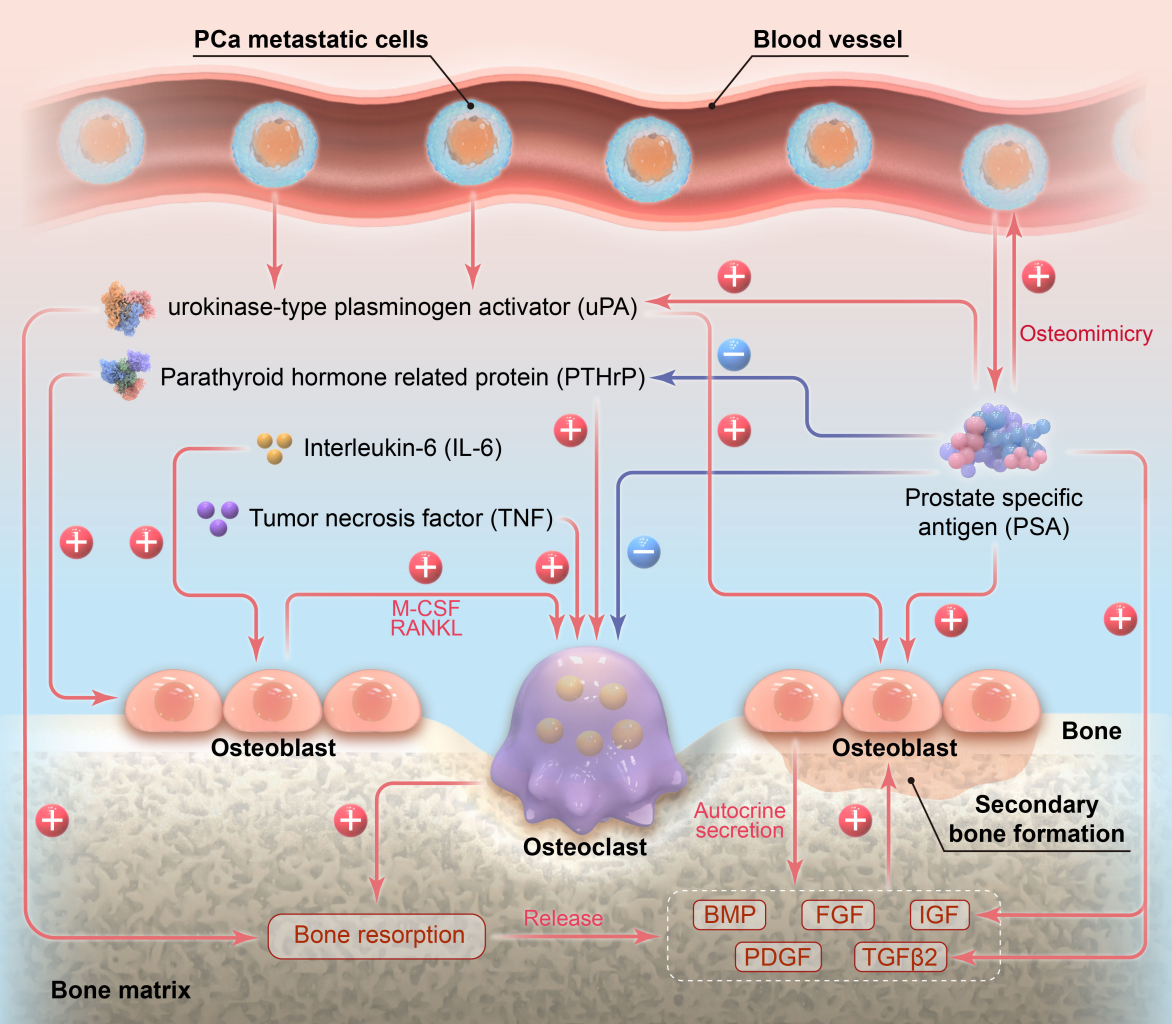
Illustration of the role of PSA in the osteoblastic bone metastasis of prostate cancer.

This review summarized the role of PSA in the osteoblastic bone metastasis and bone remodeling of PCa from two aspects: PSA-influenced cells and PSA-influenced factors involved in PCa osteoblastic bone metastasis. The related mechanisms are illustrated in **Figure 2**. Aside from PSA, another prostate-specific factor, namely prostatic acid phosphatase (PAP), can also induce the osteoblastic metastasis of PCa. PAP was once widely employed as a serum PCa marker and biochemical indicator for PCa treatment, especially for PCa with osseous metastasis, until the introduction of PSA as the new standard (69). PAP is highly expressed at PCa metastatic sites, and PAP secreted by tumor cells can stimulate OB proliferation and differentiation, and promote calcium deposit in OBs (70, 71). These effects of PAP on OBs are fulfilled in an autocrine and paracrine fashion, which modulate the balance between RANKL/OPG in favor of OPG, leading to the osteoblastic phenotype of PCa bone metastasis (72).

On top of the stimulative effects on OBs, PAP can also facilitate bone matrix formation. Phosphatase activity of PAP can generate stromal phosphate, which is fundamental to the mineralization of extracellular bone matrix (73); also, the ecto-5’-nucleotidase activity of PAP is able to generate extracellular adenosine (74), which has been reported to enhance bone matrix formation by acting on its receptors on OBs and mesenchymal stem cells (75, 76). Therefore, not only PSA but also PAP contributes to the osteoblastic feature of PCa bone metastasis, and these prostate-specific factors are all partially responsible for the difference between PCa and other malignancies regarding their phenotypes of osseous metastases.

It has been widely accepted that the effects of PSA on other biological factors mainly depend on its enzymatic activity of serine protease. Over the years, significant emphasis has been placed on documenting the enzymatically active domains of PSA and the binding sites of PSA substrates. Interestingly, Chadha et al. once reported an exception that PSA exhibited anti-angiogenic effects on human umbilical vein endothelial cells (HUVEC), and these effects were dependent on the regions outside the enzymatically active domains of PSA (77).

Despite the fact that PSA alters other biological factors by virtue of its enzymatic activity, it remains unclear how exogenous or endogenous PSA changes the phenotypes and functions of PCa cells, OCs and OBs. Apart from the aforementioned cells, PSA can also accelerate the osteogenic differentiation of mesenchymal stem cells via cadherin-Akt axis (78). Usually, biological factors exert their effects on cells by binding with corresponding membranous or intracellular receptors, and then triggering downstream signal transduction. However, as yet no such kind of receptors of PSA has been identified. Therefore, the molecular or biochemical mechanisms underlying the implications of PSA on cells stay unrevealed, and is it possible that PSA still acts on cells through its enzymatic activity? — Further studies are warranted to investigate these issues in the future.

## Author contributions

XZ collected the necessary literature, designed the whole article and finished the writing. PJ helped with data collection, article coherence, language editing and proofreading. CW proposed the ideas, corrected the mistakes and supervised the whole procedure. All authors contributed to the article and approved the submitted version.
